# Knowledge, attitudes and practices with regard to the presence, transmission, impact, and control of cystic echinococcosis in Sidi Kacem Province, Morocco

**DOI:** 10.1186/s40249-015-0082-9

**Published:** 2015-11-09

**Authors:** Ikhlass El Berbri, Marie J. Ducrotoy, Anne-Françoise Petavy, Ouaffa Fassifihri, Alexandra P. Shaw, Mohammed Bouslikhane, Franck Boue, Susan C. Welburn, Allal Dakkak

**Affiliations:** Department of Pathology and Veterinary Public Health, Institut Agronomique et Vétérinaire Hassan II, BP: 6202, Rabat, Morocco; Department of Parasitology and Medical Mycology, Lyon 1 University, 8 Avenue Rochefeller, 69373 Lyon Cedex 08, France; Division of Infection and Pathway Medicine, School of Biomedical Sciences, College of Medicine and Veterinary Medicine, The University of Edinburgh, Chancellor’s Building, 49 Little France Crescent, Edinburgh, EH16 4SB United Kingdom; Avia-GIS, Risschotlei 33, B-2980 Zoersel, Belgium; ANSES, Laboratoire de la rage et de la faune sauvage de Nancy, Laboratoire national de référence pour Echinococcus spp, 54220 Malzéville cedex, France

**Keywords:** Attitudes, Cystic echinococcosis, Knowledge, Morocco, Transmission factors, Practices

## Abstract

**Background:**

This study is a component of a large research project on five major neglected zoonotic diseases (NZDs) including cystic echinococcosis and was undertaken in the Province of Sidi Kacem over a period of four years (April 2009-March 2013).

**Methods:**

Questionnaires were administered at community level in a total of 27 communes and visits were made to all of the 10 abattoirs situated in the Province, to collect qualitative data on determinants of transmission for disease in humans and animals. More specifically, community knowledge, attitudes and practices related to cystic echinococcosis were assessed, as well as the extent to which local customs and behaviours may promote transmission. Abattoir infrastructure and practices, and their role in perpetuating disease transmission were also critically evaluated.

**Results:**

The results show that only 50 % of people have heard of the disease, and of those, only 21 % are aware of the dog’s role in disease transmission. Sixty-seven per cent of respondents stated that dogs are fed ruminant organs deemed unfit for human consumption. Owned dogs have access to the family home, including the kitchen, in 39 % of households. The extent of this close proximity between humans and animals is even more pertinent when one considers that dogs are omnipresent in the community, with an average of 1.8 dogs owned per household. The unrestricted access of dogs to abattoirs is a huge issue, which further promotes disease transmission.

**Conclusion:**

This study would suggest that the high prevalence of cystic echinococcosis in humans and animals in Morocco is largely due to three factors: 1) abundance of dogs 2) engagement in risky behaviour of the local population and 3) poor abattoir infrastructure and practices. This has serious implications in terms of the socio-economic impact of the disease, especially for rural poor communities.

**Electronic supplementary material:**

The online version of this article (doi:10.1186/s40249-015-0082-9) contains supplementary material, which is available to authorized users.

## Multilingual abstracts

Please see Additional file 1 for translations of the abstract into the six official working languages of the United Nations.

## Background

Cystic echinococcosis (CE) is a parasitic disease caused by the larval metacestode stage of *Echinococcus granulosus* (Eg). The disease has been endemic in countries of the Mediterranean region from the beginning of history [1–4]. This major neglected zoonosis has a considerable socio-economic impact including the cost of treatment of human cases and production losses in livestock. In Morocco costs associated with each surgical case have been estimated at 1500€ on average and the mortality rate of disease at 2–3 % [5, 6]. When this cost is multiplied by the average of 5.1 surgical cases per 100,000 inhabitants recorded in 2003–2010 [7], the economic burden from human cases alone is substantial.

Morocco has one of the highest reported incidence rates of CE in North Africa. Indeed, in Eastern Libya, a surgical incidence rate of 4.2 cases per 100,000 inhabitants was reported [8] while 36 surgical cases were recorded in 2008–2011 in the west of the country [9]. In Egypt, an annual rate varying between 1.3 and 2.6 cases per 100,000 inhabitants was reported [10]. In Algeria, official surveys (2000–2008) showed an annual surgical incidence varying between 1.3 and 2.5 cases per 100,000 inhabitants [11]. However, in Tunisia a higher incidence of 12.6 human cases/100,000 inhabitants was reported in 2001–2005 [12]. It must be emphasized that these reported cases represent the ‘tip of the iceberg’ as many cases go undetected.

In livestock, in addition to production losses ante mortem, costs associated with condemnation of infected organs (liver and lungs) increase the economic burden of the disease. The overall cost to both the human health and livestock sector is estimated at nearly 1,000,000€/year [13].

The worldwide annual socioeconomic impact of cystic echinococcosis has been estimated at 1,009,662 DALYs and US $763,980,979. A global annual livestock production loss of at least US $141,605,195 and possibly up to US $2,190,132,464 is also estimated [14]. Collectively, parasitic zoonoses (of which echinococcosis is a major contributor) probably have a similar human disease burden to any one of the big three human infectious diseases: malaria, tuberculosis or HIV in addition to animal health burden [15]. An update on the global burden of foodborne parasitic diseases indicates that parasitic diseases that can be transmitted through food make a substantial contribution to the global burden of disease [16].

The main objectives of the study were to determine the main attitudes and practices that promote the transmission of CE. A large-scale questionnaire survey was undertaken in the Province of Sidi Kacem to firstly evaluate community knowledge on mode of transmission of CE in humans and animals, and secondly to identify the most important routes of infection for ruminants, dogs and humans in this context. Sidi Kacem Province is a good case study area as prevalence/incidence of CE has been reported to be high both in animals (prevalence of 42.9, 11.0 and 1.5 % in cattle, sheep and goats respectively) [17] and humans (incidence of 6.5 surgical cases per 100,000 inhabitants) [7], and its geographic and climatic diversity makes it a model representative of many parts of the Kingdom.

Sidi Kacem Province is subdivided into two zones, referred to as ‘rainfed’ (*bour*) and ‘irrigated’ *(irrigué)*, which are distinct with regard to hydrologic but also demographic, agro-ecological and socioeconomic characteristics (Table 1). The rainfed zone extends from the hilly northeast border of the Province to the centre and the irrigated zone occupies the flatter terrain from the centre to the southwest. The number of inhabitants, cattle population and sheep population in the irrigated zone is almost double that in the rainfed zone. More goats are kept in the rainfed zone, as this species is more adapted to mountainous terrain. There are more cattle keepers in the rainfed zone, although the cattle herd size is much smaller and livestock production more extensive in character than in the irrigated zone. Small ruminant herd size is similar for the rainfed and irrigated zone [18]. Although no data contrasting poverty indices and level of education across the two zones are available, the northern rainfed zone is regarded as being much poorer (households have a lower socio-economic status) and has a higher illiteracy rating than the irrigated zone [19].

**Table 1:** Comparison of demographic and agro-ecological characteristics of rainfed and irrigated zones of Sidi Kacem [18]

Characteristic	Rainfed (%)	Irrigated (%)	Overall
Number of communes	12 (41.4)	17 (58.6)	29
Number of douars (villages)	349 (45.7)	415 (54.3)	764
Number of inhabitants	159,202 (32.4)	332,395 (67.6)	491,597
Number of cattle keepers	8,866 (65.3)	4,707 (34.7)	13,573
Number of small ruminant keepers	5,662 (50.0)	5,655 (50.0)	11,317
Cattle population (estimate)	48,000 (40.1)	71,840 (59.9)	119,840
Sheep population (estimate)	167,130 (44.1)	212,100 (55.9)	379,230
Goat population (estimate)	11,360 (54.7)	9,410 (45.3)	20,770
Dominant livestock production system	Extensive	Intensive	NA
Average cattle herd size	5	15	9
Average small ruminant flock size	32	39	35
Altitude (metres)	150-500	50-150	NA
Topology	Hills and hillocks	Flat open country	NA

NA, not applicable

## Methods

The present study is a component of a large collaborative project, ICONZ (Integrated Control of Neglected Zoonoses) funded by the EU under FP7. This project aims at improving human health and animal production in developing countries through Integrated Control of Neglected Zoonoses in animals, based on scientific innovation and public engagement. In Morocco the project includes, besides cystic echinococcosis, four other major neglected zoonotic diseases (NZDs), namely: Bovine Tuberculosis, Brucellosis, Leishmaniasis and Rabies. The study was undertaken in the Province of Sidi Kacem.

### Study design

Sidi Kacem Province was chosen as a case study site because of its large dog, cattle, sheep and goat populations, estimated at 20,800, 120,000 and 380,000 and 21,000 respectively (Table 1).

**Fig. 1:**
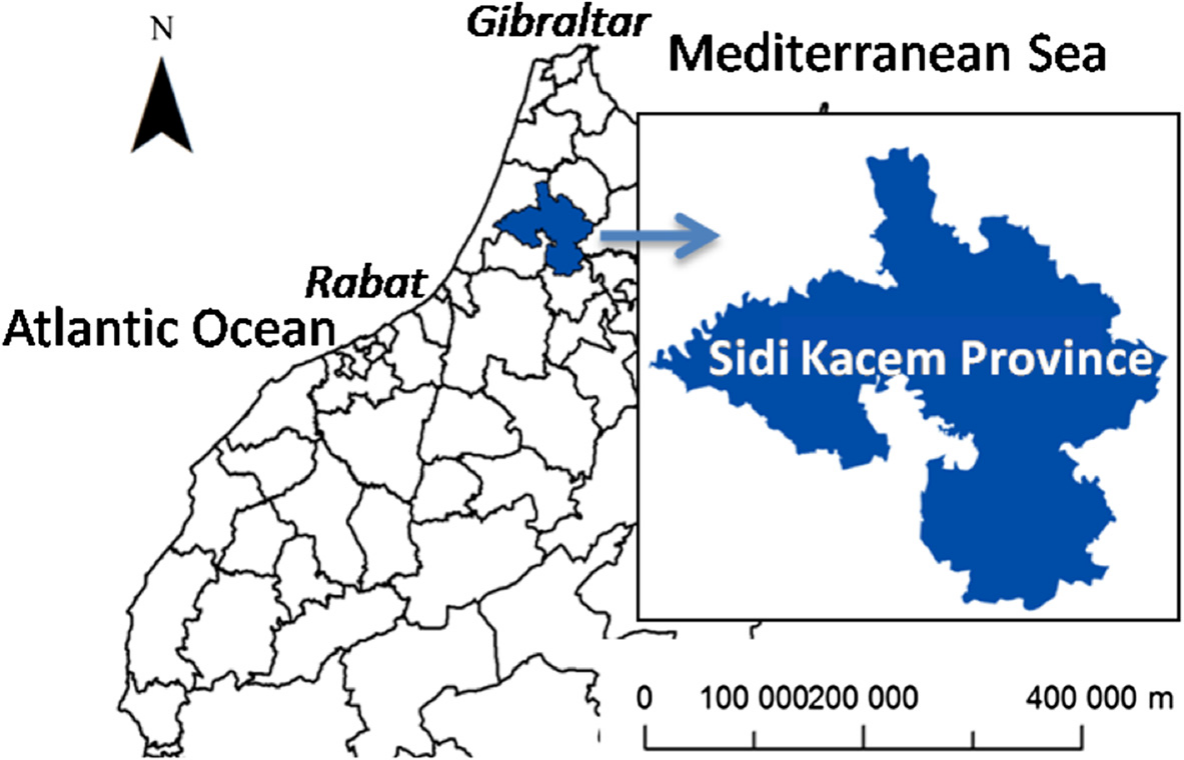
Map of Morocco showing the location of the Province in the northwest of Sidi Kacem

Sidi Kacem is located in the Northwest of Morocco (Fig. 1) and the rate of poverty in the Province is estimated at 14.9 % versus the 9 % national average [18]. Before the ICONZ study very little information had been available on CE for this Province. The present study spans four surveys in Sidi Kacem Province over four successive years (2009–2013). In total, 27 communes out of 29 were targeted by the study, as well as all 10 abattoirs of the Province.

## Methodology

### Data collection

The main objective of the study was to assess local community knowledge of CE and the impact of socio-economic, cultural and environmental factors on its mode and extent of transmission. The study involved two components: 1) a community questionnaire survey and 2) an abattoir study.

#### Community questionnaire survey

A locally validated questionnaire was administered to 543 community members in 39 *douars* (villages) across the 27 communes studied. For each of the 27 communes, one to two *douars* were randomly selected from the list of villages in each commune obtained from the *caid* (an appointed commune government official). At village level, between 5 and 20 households were randomly selected from lists of households in each village obtained from the *sheikh* (village head). The vast majority of interviewees were male (>90 %) and 453 (83 %) of households in the study owned dogs.

#### Abattoir study

The 10 abattoirs of Sidi Kacem Province were visited and informal key informant interviews (KII) were undertaken with one to two abattoir personnel per abattoir. In total, 15 abattoir workers were interviewed. During abattoir visits, the abattoir infrastructure as well as the behaviour of butchers and abattoir personnel were observed and evaluated.

Collection of information by questionnaire or KII was undertaken on an entirely voluntary basis and was conducted by trained enumerators. Verbal consent was obtained from all study participants.

### Statistical analysis

We investigate significant differences between knowledge/attitudes/practices (KAP) across two parameters: 1) household location in rainfed versus irrigated zone, and 2) livestock keeping versus non-livestock keeping household, for 120 households interviewed in 2012–2013. Comparison of proportions across variables was firstly undertaken by calculating 95 % confidence intervals using Fisher’s exact method in WinPepi© [20]. Overlap in confidence intervals essentially means that no conclusion can be drawn with regard to differences between categories [21]. Proportions were compared in WinPepi© through calculation of Fisher’s odds ratio (and Fisher’s exact 95 % confidence interval) and Pearson’s chi-square test [22]. Analysis of variance was used to assess whether there was a difference in continuous KAP variables. The Kruskal-Wallis one-way ANOVA was used because within-group deviations (residuals) of data did not follow a Normal distribution [23] and was computed in Minitab [24].

## Results

### Investigations with rural population

#### Household socioeconomic characteristics

**Fig. 2:**
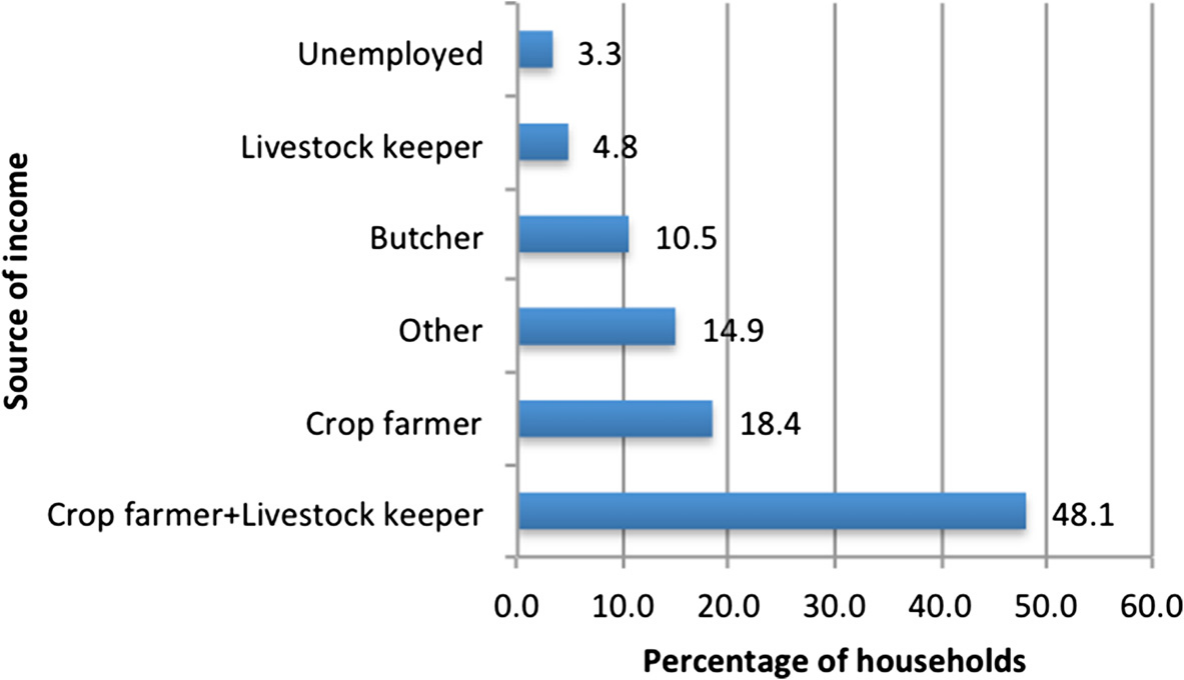
Percentage of households that derive income from specific activities

Crop farming and livestock production are the two main sources of income for Sidi Kacem households, with 71.3 % of persons being engaged in either or both activities (Fig. 2). Education levels are below average, with only 1.7 of the 2.4 children per household attending school, on average.

#### Evaluation of knowledge, attitudes and practices related to dog ownership and CE

**Fig. 3:**
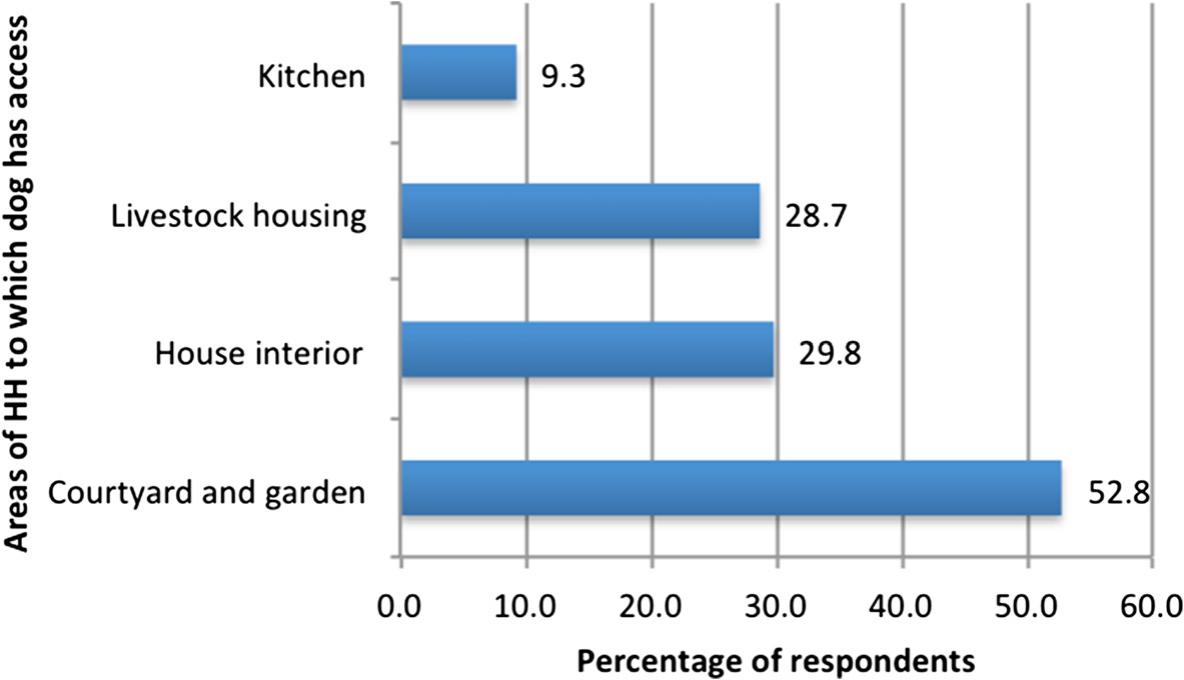
Percentage of respondents reporting access of dogs to specific areas of the homestead

Evaluation of community knowledge showed that out of 543 persons interviewed, only 50.3 % have heard of CE. Only 21.3 % of 423 respondents are aware of the dog’s role in disease transmission. The average number of dogs owned per household is 1.8. Forty-two out of 453 (9.3 %) persons interviewed give their dog(s) access to the kitchen, 29.8 % allow their dog(s) to roam around the family home and 28.7 % confirmed that their dog(s) have access to livestock housing. However, 52.8 % of persons claimed that dog roaming is restricted to the household courtyard and garden (Fig. 3).

Furthermore, 71.7 % of those 453 respondents report encouraging their dogs to roam beyond the family home, including 34.4 % reporting that their dog is entirely free to roam anywhere and 7.1 % who specified that their dog is encouraged to frequent the *souk* (livestock market) and abattoirs. In Morocco the *souk* takes place on a weekly basis in every small town.

Two hundred and thirty-eight (43.2 %) of the 539 respondents own sheepdogs, and only 4.6 % report deworming, with only 1.5 % doing it regularly (every three to four months). Among 448 persons questioned, 70.0 % note the presence of stray dogs in their *douar*, with an average of 5 stray dogs per *douar* (calculated by averaging the total number of stray dogs reported to be present across the number of interviewees from that *douar*).

#### Evaluation of CE transmission risk to humans with regard to community behaviour

**Fig. 4:**
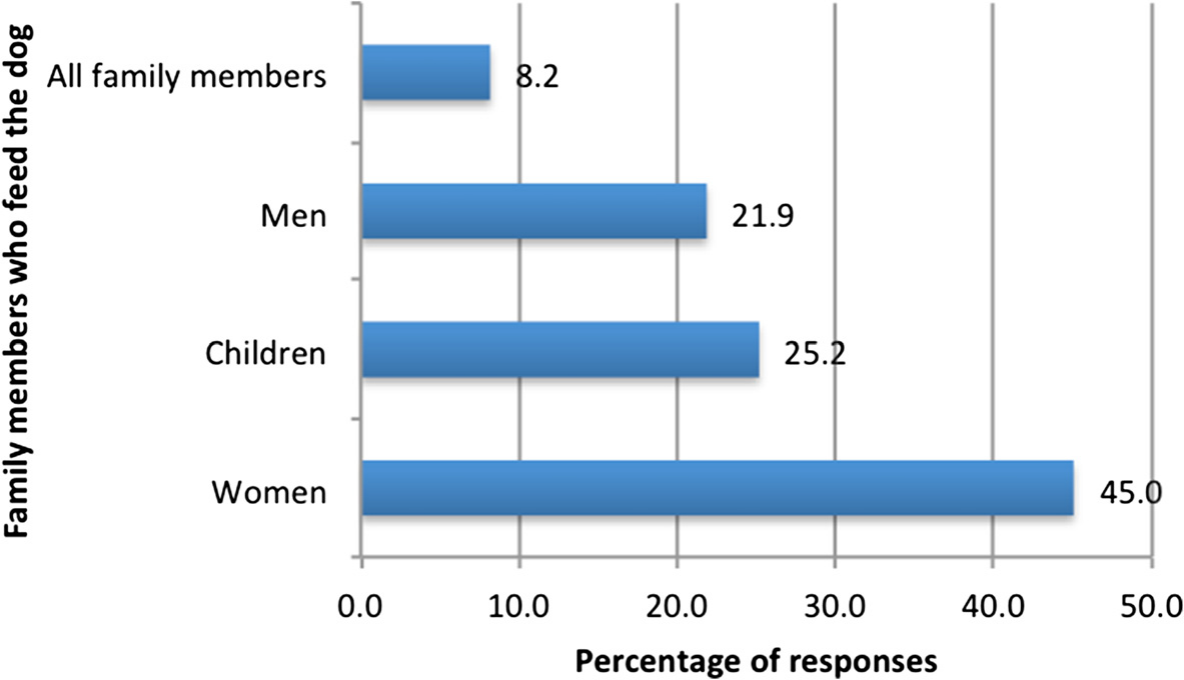
Percentage of responses on persons responsible for dog feeding

Among 407 respondents, 61.2 % reported that their children do not wash their hands after petting or playing with dogs. Furthermore, for 453 people surveyed, 45.0 % report that it is the women who are mainly in charge of dog feeding, with only 21.9 % reporting that men feed dogs, and 25.2 % reporting that it is the children’s responsibility. Thirty-seven (8.2 %) households report that all family members take turns to feed the family dog (Fig. 4).

**Fig. 5:**
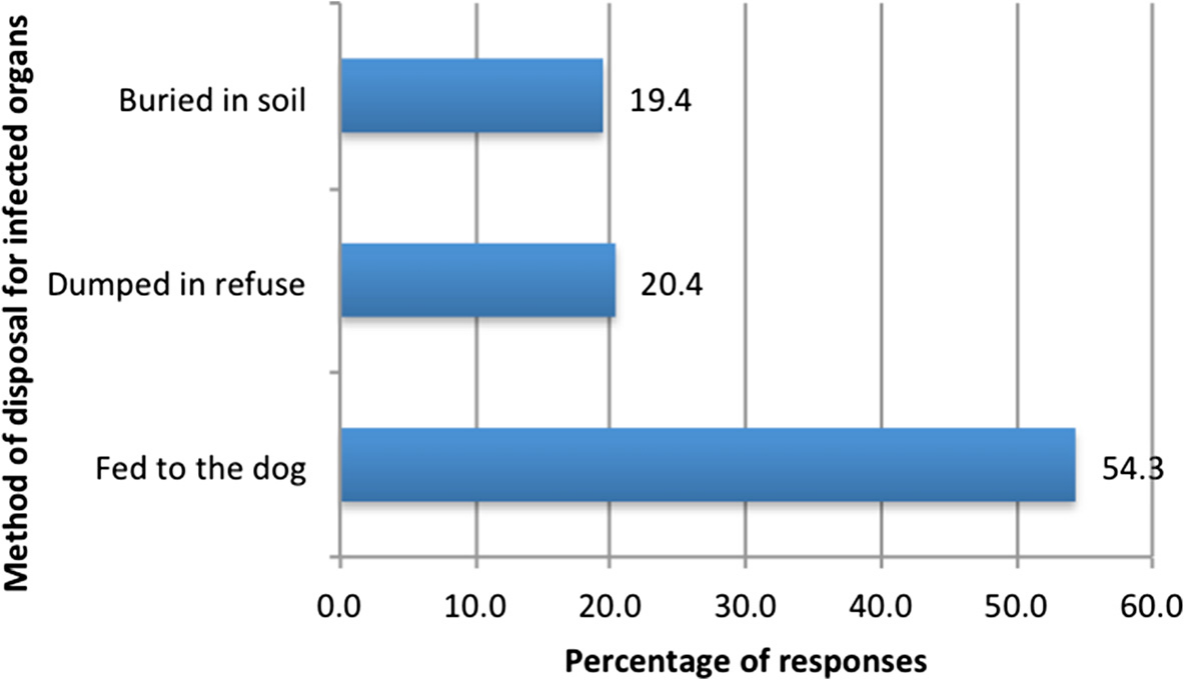
Percentage of responses on method of disposal of infected organs

Moreover, 54.3 % of the 543 respondents feed organs (predominantly liver and lungs) recognised as infected and inappropriate for human consumption to their dog(s). Fifty-seven (20.4 %) of 280 people, dispose of infected liver and lungs as refuse thrown in outdoor dumping grounds and therefore accessible to owned and stray dogs. It is important to emphasise that there is no roadside collection of household waste in Sidi Kacem. In contrast just 19.4 % of 453 persons report burying infected organs to prevent their consumption by domestic carnivores (Fig. 5).

Only 31.1 % of 543 households questioned during the survey are supplied with treated and safe ONEP (Office National de l’Eau Potable) water, while 68.9 % households still use traditional water sources, namely wells, cisterns and streams/rivers. In addition, 88.2 % of 543 respondents report consumption of raw vegetables purchased from *souks* (markets), harvested from fields, or gathered from their personal vegetable patch, and some persons even claim to consume wild plants.

A summary of the main risk factors for CE transmission in Sidi Kacem province is presented in Table 2.

**Table 2:** Assessment of major risk factors for CE transmission in Sidi Kacem province

Knowledge, attitudes and practices	% of respondents (n)
Ignorance of the disease	49.7 (543)
Ignorance of the dog’s role in disease transmission	78.7 (423)
Dog has access to the family home	39.1 (453)
Dog encouraged to roam freely	34.4 (453)
Children do not wash their hands after petting or playing with dog(s)	61.2 (407)
Feeding of infected organs to dog(s)	54.3 (543)
Use of traditional water sources	68.9 (543)

### Human hydatidosis in the Province of Sidi Kacem

A retrospective study (2003–2010) in the Province of Sidi Kacem revealed that 269 persons had surgery to remove hydatid cysts during this period, giving an average annual incidence of 6.5 surgical cases per 100,000 inhabitants. Furthermore, a predominance of cases in females and cases from rural areas was observed, namely 77.0 and 77.0 % in 2008, and 78.9 and 84.2 % in 2010 respectively [7].

### Evaluation of abattoir infrastructure and practices

The infrastructure and hygiene practices in Sidi Kacem abattoirs were found to be highly unsatisfactory from the perspective of mitigating transmission. Abattoirs are generally composed of a simple slaughter hall where all slaughter operations, including evisceration, are conducted. None of the abattoirs visited had refrigeration equipment; meat is thus transported and, in most cases, consumed the day of slaughter without respecting the requirement for a period of maturation. Furthermore, none of the ten abattoirs have a receptacle for discarding of condemned organs and therefore dogs have easy access to infected organs. Dogs were observed to roam around the slaughterhouse in packs, and sometimes even entered the slaughter hall. Informal discussions with butchers and abattoir personnel revealed that presence of dogs within the slaughter hall is tolerated and even encouraged due to the dog’s role in consuming organs that have been discarded due to being recognised as infected with echinococcosis cysts. Dogs are therefore regarded as a form of free waste disposal and a way to ‘tidy up’ the abattoir.

### Differences in KAP across rainfed/irrigated zone and livestock and non-livestock keeping households

Table 3. indicates that a significantly higher proportion of households in the rainfed zone of Sidi Kacem 1) notice liver and/or lung lesions during home slaughter (*p* < 0.1); 2) drink potentially contaminated water from traditional sources (*p* < 0.01) and 3) have heard of CE (*p* < 0.05). The proportion of households reporting that dogs and cats from outside (strays or neighbour’s pets) consume infected organs is significantly higher in the irrigated zone (*p* < 0.05). A significantly higher proportion of non-livestock keeping households feed infected organs to dogs than livestock keepers (*p* < 0.05). Interviewees from livestock keeping households are more likely to know someone with CE (*p* < 0.1).

**Table 3:** Difference between knowledge, attitudes and practices (categorical variables) versus household location in rainfed/irrigated zone of Sidi Kacem Province and livestock keeping versus non-livestock keeping households

*Variable*	*No. HH (yes)*	*% yes (95 % CI)* ^*a*^	*Odds Ratio (95 % CI)* ^*a*^	*Chi-square (DF, p)* ^*b*^
*Liver/lung lesions noticed during home slaughter*				
Rainfed	71 (43)	60.56 (48.25–71.97)	1.88 (0.85–4.21)	2.866 (1, 0.090)*
Irrigated	49 (22)	44.90 (30.67–59.77)		
*Dogs and cats from outside consume infected organs*				
Irrigated	49 (42)	85.71 (72.76–94.06)	3.91 (1.45–11.65)	8.877 (1, 0.003)**
Rainfed	71 (43)	60.56 (48.25–71.97)		
*Household drinks potentially contaminated water (water other than from tap)*				
Rainfed	71 (67)	94.37 (86.20–98.44)	6.70 (1.89–29.58)	11.964 (1, 0.001)***
Irrigated	49 (35)	71.43 (56.74–83.42)		
*Responder has heard of CE*				
Rainfed	71 (44)	61.97 (49.67–73.24)	2.81 (1.24–6.40)	7.394 (1, 0.007)**
Irrigated	49 (18)	36.73 (23.42–51.71)		
*Feeding of infected organs to dog(s)*				
Non-livestock keeping	29 (22)	75.86 (56.45–89.70)	2.82 (1.02–8.53)	4.834 (1, 0.028)**
Livestock keeping	91 (48)	52.75 (42.00–63.31)		
*Responder knows someone with CE*				
Livestock keeping	91 (25)	27.47 (18.63–37.83)	3.28 (0.88-18.28)	3.606 (1, 0.058)*
Non-livestock keeping	29 (3)	10.34 (2.19–27.35)		

(^a^By Fisher’s method; ^b^Pearson’s chi-square; statistically significant at: 10 % level*; 5 % level**; 1 % level***)

It must be emphasized that overlap of confidence intervals between categories occurs for all response variables except 1) dogs and cats from outside consuming infected organs and 2) households drinking water other than tap water, limiting the drawing of firm conclusions with regard to differences between categories to these two variables only. This is reflected in the *p* values, which are lowest for these two variables. The highest probability of making a Type I error (rejecting the null hypothesis when it is true) applies to the variables ‘liver/lung lesions noticed during home slaughter’ and ‘responder knows someone with CE’, and for both variables the lower 95 % confidence limit of the odds ratio is below 1, and the chi-square test is statistically significant at the 10 % level only.

Table 4 shows that 1) the distance of study households from the nearest abattoir and 2) the number of stray dogs in the neighbourhood as reported by the interviewee is significantly higher in households of the rainfed than irrigated zones (*p* < 0.01). The number of dogs owned by livestock keeping households is also significantly higher than in non-livestock keeping households at the 1 % level.

**Table 4:** Difference between knowledge, attitudes and practices (continuous variables) versus household location in rainfed/irrigated zone of Sidi Kacem Province and livestock keeping versus non-livestock keeping households

*Variable*	*No. HH*	*Median*	*Kruskal-Wallis Test (adjusted for ties)*	
			*H*	*p*
*Distance of household from abattoir*				
Rainfed	71	11.0	27.32	0.000
Irrigated	49	2.0	(27.67)	(0.000)
*Number of stray dogs in neighbourhood*				
Rainfed	71	2.0	8.34	0.004
Irrigated	49	5.0	(8.75)	(0.003)
*Number of dogs owned per household*				
Livestock keeper	91	2.0	6.32	0.012
Non-livestock keeper	29	1.0	(7.41)	(0.006)

## Discussion

### Risk factors favouring CE infection in animals

Agriculture is a priority for the Province of Sidi Kacem and the main contributor to the economy, with 70.4 % of the total surface area being dedicated to this activity. The abundance of pasture, which is the main source of nutrition for ruminants in this setting, is dependent on the favourable terrain and climate: existence of plains, adequate rainfall (600 mm) and water resources (five of the country’s main rivers flow through this region). Pastures are unfortunately accessible to dogs, as confirmed by 34.4 % of respondents. Furthermore, 43.2 % of the population own sheepdogs and 28.7 % persons report that their dogs have regular access to livestock housing, where they can thus contaminate the animal’s feeding and drinking water with Eg eggs. Moreover, according to 7.1 % respondents, dogs frequent abattoirs on market day, where they invariably become infected with Eg considering the neglectful practices of slaughterhouse workers and inadequate infrastructure of abattoirs. This finding is in agreement with other studies conducted in other regions of the country, namely in Rabat [25], in Ouarzazate [26] and in Tetouan [27], where abattoirs, especially in rural area, were considered as the main source of dog infection. A study by Bardosh et al. [28] undertaken in Sidi Kacem Province concluded that social, infrastructural, economic and political determinants are entwined at slaughterhouses in Morocco, normalising the open disposal of hydatid cysts to free roaming dogs. Surveys conducted by Van Kesteren et al. [29] in Kyrgyzstan revealed that many households had dogs and that dogs played various roles in the communities, as pets, guard dogs or sheep dogs. Almost all dogs were free to roam, and GPS data revealed that many moved outside their communities, thus being able to scavenge offal.

The extent of infection is aggravated by the fact that only 1.5 % of respondents deworm their dogs, and 54.3 % of respondents feed them infected organs. These findings corroborate those reported by Azlaf and Dakkak [30] in other regions of Morocco as well as Benabid et al. [31] in Tunisia. Similarly, a study conducted in Eastern Algeria found that 91.1 % of rural households did not deworm their dogs, although the percentage of households feeding dogs infected liver and lungs was found to be lower at 12.1 % [32].

The co-existence of transmission risks in this context, namely a livestock keeping and dog owning community, promotes the infection of both ruminants and dogs in tandem. Livestock, a very important component of rural community livelihoods (52.9 % of population are livestock rearers), thereby becomes a principal risk factor for the disease through persistent generation of infected viscera (as a result of animal deaths at pasture, household-level religious slaughter during the Aid al-Adha, or abattoir slaughter). Livestock in this setting represents a constant source of protoscoleces, which are easily accessible to dogs for the aforementioned reasons.

The dog is omnipresent in the community with households owning an average of 1.8 dogs, and this does not take into consideration the stray dog population. Moreover 70.0 % of respondents confirmed the presence of stray dogs within their *douar* of residence. The remaining 30.0 % did not report personally observing stray dogs in their *douar*, but when further questioned remarked that they had not paid much attention to this issue. Often these persons were from the same *douar* as other persons who confirmed observing stray dogs. This value (70.0 %) is much higher than the 38.1 % of households in a rural area of Algeria reporting presence of stray dogs in their neighbourhood [32], emphasising the extent of the stray dog problem in Sidi Kacem.

With widespread presence of infected ruminants and roaming behaviour of a large dog population, it is unsurprising that a recent survey in owned dog of Sidi Kacem reported an Eg prevalence of 35.3 % (after arecoline purgation) [33]. Ruminants can become infected from pasture or in livestock housing through ingestion of forage, feed or water contaminated by parasitized dogs’ faeces. The data collected during this study suggests that contamination is widespread given the uncontrolled roaming of dogs, which was also identified as a risk factor of disease transmission for ruminants in other regions of Morocco [34, 35].

Livestock has been reported as a major risk factor for CE in multiple other studies in China [36, 37], Tunisia [31] and Peru [38]. However, it should be emphasized that whilst disease distribution is closely linked to zones where livestock is kept, some authors report that there is no correlation between animal density and disease frequency [39].

### Risk factors promoting human infection

A retrospective study (2003–2010) of human CE in the Province of Sidi Kacem, showed a high prevalence of this disease in the region, with an annual average incidence of 6.5 surgical cases per 100,000 in habitants compared to the national average of 5.1 surgical cases per 100,000 in habitants recorded in the same period. This prevalence varies according to the origin and sex of patients. Indeed, 80.6 % of cases come from rural areas and 78.0 % of cases occur in women [7].

Results from our survey provide some explanations as to why women and rural communities may be more at risk of the disease. It is mainly women who have the responsibility of feeding dogs (45 % of respondents) and they therefore have greater contact with dogs making them more exposed to infected dog faeces. This finding has been observed in various other regions of Morocco by Mahjour et al. [40] and Ouhelli et al. [41] in different areas, by Achemlal [27] in the region of Tetouan, by Tabyaoui [42] in the region of Azrou and El Mansouri et al. [43] in the region of Rabat. This was also observed by authors in other countries including Wales [44], Iraq [45], Jordan [46], in Turkey [47], China [36, 37] and Tunisia [48].

Despite children being the next most frequent group to feed dogs after women (25.2 % of responses), it is important to note the low national CE incidence rate noted in this age group (10.0 %) [49]. This is due mainly to the prolonged latency period of CE before clinical presentation [50].

The over-representation of cases from rural communities is also due to the dominance of rural communities in the province (71.0 %) and the fact that people are heavily dependent on livestock which is, as mentioned above, a potential source of transmission to dogs, and ultimately humans. Household members working in animal husbandry was associated (*p* < 0.05) with a risk of having at least one family case of hydatidosis in rural households in Algeria, highlighting the link between livestock ownership/contact and human disease. This same study found that 4.6 % of urban households and 14.6 % of rural households reported at least one case (*p* < 0.001) [32].

Another factor is the limited education and income of the rural population: 27.9 % of households have children that do not attend school. The province of Sidi Kacem is among the poorest in the Kingdom with a poverty rate of 14.9 % as compared to the national average of 9 %. The socioeconomic situation has been reported as a risk factor for CE transmission in other countries such as China [36, 37].

The close proximity between dogs and humans promotes human infections: 9.3 % of respondents reported that their dogs have access to the kitchen, increasing the risk of food contamination with infected dog faeces. Moreover, 29.8 % of owned dogs have free access to the family home. Thus, a high risk of human disease exists especially in rural areas where the dog is omnipresent. In Algeria, possession of more than one dog was associated with hydatidosis (*p* < 0.1) in urban areas. Moreover, presence of stray dogs in the district was also found to be associated with the disease [32].

Poor community knowledge of transmission risks perpetuates transmission as only 50.3 % respondents are aware of the disease, and only 21.3 % recognise the dog’s role in disease transmission to humans and domestic animals. This is reflected in engagement in risky behaviour such as mismanagement of infected ruminant organs and poor hygiene practices, especially amongst children. Indeed, 61.2 % of persons admitted that their children do not wash their hands after petting or playing with dogs, increasing the risk of exposure to the disease.

Similar results have been reported by Khayat [51] and Tair [52] in the regions of Khenifra and Ouezzane respectively in Morocco, and Oudni et al. [53] in Tunisia. In Algeria, unfamiliarity of the disease was found to be less marked than in this study, with 91.7 and 87.4 % of households in urban and rural areas respectively being aware of hydatidosis (at least by name).

In addition, 68.9 % of surveyed households drink potentially contaminated water from wells, streams and rivers, and 88.2 % consume raw vegetables. These two practices combined with the uncontrolled roaming of dogs and dissemination of Eg eggs in fields, pastures and water resources, constitute a major risk factor for human disease. This finding corroborates the findings of other studies undertaken in Morocco, namely those undertaken in the region of Loukkos [35, 54] and in the region of Khenifra [34], as well as others studies undertaken in Uruguay [55], Jordan [56], Argentina [57] China [36] and Tunisia [31].

### Differences in KAP between rainfed/irrigated zones and livestock keeping versus non-livestock keeping households

The higher proportion of households noticing liver and/or lung lesions during home slaughter in the rainfed (60.6 %) as compared to the irrigated (44.9 %) zone could be due to the fact that inhabitants of the rainfed zone are poorer and do not have the financial means to treat their livestock. They may also be more likely to slaughter older ruminants, based on their lower price. As infection rate increases with age of host [17], there may be a higher probability of households slaughtering animals with lesions in the rainfed zone. Alternatively, the fact that a higher proportion of respondents from the rainfed zone have heard of the disease may promote the ability to recognize lesions during home slaughter. Hence infection rate could be constant across the two zones but appear higher in the rainfed zone based on a higher index of suspicion of the householders. Better knowledge of CE in the rainfed zone could be related to over-representation of the disease in this zone (Table 3).

The higher median of stray dogs in neighbourhoods of the irrigated zone could in part explain the reason why respondents from this zone are more likely to report consumption of infected organs by outside dogs. The more highly populated irrigated zone can hypothetically support a higher population of stray dogs, which may increase the disease risk in this zone. The fact that households in the irrigated zone are situated in closer proximity to abattoirs (both formal and informal) is also of concern, as this promotes dog infection through access to abattoir waste, which as previously mentioned is a widespread problem in Sidi Kacem [28] (Table 4).

A major limitation of comparing KAP across rainfed and irrigated zones is that they represent contrasting characteristics and variables which themselves could account for the differences observed in KAP, making the concept of ‘hydrological zone’ a confounder. Unfortunately, data on other variables of interest such as household wealth status, stray dog density etc. was beyond the scope of this study but we have been able to compare KAP variables between livestock keeping and non-livestock keeping households.

Interestingly, a higher proportion of non-livestock keeping households feed infected organs to dogs, which could relate to the finding that respondents from livestock keeping households are more likely to know someone with CE. The rationale for this link is that livestock keeping communities or areas where livestock keeping dominates may be correlated with a higher risk of human infection. In areas where there are more sufferers of the disease, awareness can be predicted to increase. Although the difference was not found to be statistically significant, respondents from livestock keeping households were found to be more knowledgeable of CE. A study undertaken in Algeria also found that a higher proportion of households affected by hydatidosis had knowledge of CE as compared to non-affected households. The explanation given is the ‘educational impact’ of being personally confronted with the disease [32]. Family cases of human CE may generate better knowledge of the disease in rainfed zones, and better understanding of the link between the manifestation of disease in livestock and human disease could in part explain why a lower proportion of households from the rainfed zone feed infected organs to dogs (Table 3).

The number of dogs owned per household was found to be higher in livestock keeping households. Livestock keeping households own more dogs because they rely on dogs to guard and herd livestock. This correlation between livestock and dog ownership is a double risk factor for successful completion of the parasite lifecycle through co-habitation of intermediate and definitive hosts (Table 4).

Hydrological zone as a confounder for other variables is possible for all variables investigated except for ‘consumption of water other than from tap’. It is logical for households situated on irrigated land to also have better access to ONEP tap water than households in the rainfed zone, although the proportion of households that drink potentially contaminated water is remarkably high even in the irrigated zone (71.4 %) (Table 3).

## Conclusion

CE in Morocco is a serious burden disproportionally borne by poor, rural and livestock keeping communities. In Sidi Kacem Province 19 surgical cases of CE were reported in 2010, 84.0 % of which were from rural communes [7]. The infection rate in slaughter animals was found to be very high, at 42.9, 11.0 and 1.5 % for cattle, sheep and goats respectively [17]. The prevalence of Eg in owned dogs of Sidi Kacem is equally concerning at 35.3 % [33].

The main drivers for CE transmission in Sidi Kacem Province have been identified as follows:

- The uncontrolled roaming of owned and stray dogs, the close proximity and co-habitation of owned-dogs with their owners and the infrequent practice of dog deworming;
- The traditions and practices of the local population (home slaughter, consumption of raw vegetables, consumption of untreated water from wells, etc.),and poor hygiene practice especially in children;
- Abattoirs, which are a source of a huge volume of contaminated organs, to which free-roaming dogs have easy access due to inadequate disposal of condemned material.

Active surveillance of CE has been undertaken in Morocco since the establishment of the One Health platform, the National Interministerial Committee for the control of Hydatidosis (Comité National Interministériel de Lutte contre l’Hydatidose). At the Provincial level, the Committee is responsible for the application of control measures and for monitoring the evolution of disease, but this is heavily focused on human disease. Animal cases detected during routine abattoir inspection are unfortunately not reported. This work has identified the drivers for disease transmission and sets a clear agenda and priorities for controlling the disease in Sidi Kacem province. Morocco has the platform to roll-out inter-sectoral and integrated control strategies, now it is time for political will to follow and for the Interministerial Committee to be given the means and resources to put this knowledge into use.

## Additional file

Additional file 1:
**Multilingual abstracts in the six official working languages of the United Nations.** (PDF 552 kb)

## Abbreviations

CE: Cystic Echinococcosis
DALYs: Disease-Adjusted Life Years
EU: European Union
Eg: Echinococcus granulosus
HIV: Human immunodeficiency virus
IAV HII: Institut Agronomique et Vétérinaire Hassan II
ICONZ: Integrated Control of Neglected Zoonoses
KAP: Knowledge/Attitudes/Practices
KII: Key Informant Interviews
NZDs: Neglected Zoonotic Diseases
ONEP: Office National de l’Eau Potable
