# Supplementary material for: Knowledge, attitudes and practices with regard to the presence, transmission, impact, and control of cystic echinococcosis in Sidi Kacem Province, Morocco
Source: Infect Dis Poverty. 2015 Nov 9;4:48. doi: 10.1186/s40249-015-0082-9 (PMC4638086; doi:10.1186/s40249-015-0082-9)
Supplement: Additional file 1: — Multilingual abstracts in the six official working languages of the United Nations. (PDF 552 kb) [file 40249_2015_82_MOESM1_ESM.pdf]

Translation of the abstract into the six official working languages of the United Nations

المعرفة والسلوكيات والممارسات ذات الصلة بوجود داء المشْوكات الكيسية، وانتقاله وتأثيره في مقاطعة سيدي قاسم، المغرب

البربري إخلاص[a]، دوكروتوي ماري جولي[c*] ، بيتافي آن فرانسواز[b]، الفاسي الفهري وفا[a]، شو ألكسندرا[c,d]، بوصليخان محمد[a]، بو فرانك[e]، سو ويلبرن[c]، دقاق علال[a]

الملخص

الخلفية: هذه الدراسة تتألف من مشروع بحثي كبير على خمس أمراض رئيسية مهملة حيوانية المصدر تتضمن داء المشْوكات الكيسية وتم إجراؤها في مقاطعة سيدي قاسم على مدى 4 سنوات (أبريل 2009 – مارس 2013).

الطرق: كانت الاستبيانات تطرح على مستوى المجتمع المحلي في ما مجموعه 27 بلدية و أجريت الزيارات لكافة المسالخ العشرة االموجودة في المقاطعة لجمع البيانات النوعية الخاصة بمحددات انتقال للمرض لدى البشر والحيوانات. وبشكل أكثر تحديدا، تم تقييم المعرفة المجتمعية والسلوكيات والممارسات ذات الصلة بداء المشْوكات الكيسية، إضافة إلى المدى الذي يمكن فيه للعادات والسلوكيات المحلية تعزيز الانتقال. كما أجري تقييم صارم للبنية التحتية للمسالخ والممارسات، ودورها في إدامة انتقال المرض.

النتائج: أظهرت النتائج أن 50% فقط من الناس قد سمعوا عن هذا المرض، وأن21٪ فقط من بين هؤلاء يدركون دور الكلب في انتقال المرض. وذكر سبع وستون في المائة من الذين استجابوا للاستبيان أن الكلاب يتم إطعامها أعضاء الحيوانات المجترة التي تعتبر غير صالحة للاستهلاك البشري. الكلاب المملوكة يمكنها الدخول إلى منزل الأسرة، بما في ذلك المطبخ، لدى 39% من الأسر. وتزداد أهمية هذا التقارب بين البشر والحيوانات عندما يضع المرء في اعتباره مدى انتشار الكلاب في كل مكان في المجتمع، بمعدّل قدره 1.8 كلبا تمتلكه الأسرة الواحدة. إن الوصول غير المقيد للكلاب إلى المسالخ قضية كبيرة، مما يزيد في تعزيز انتقال المرض.

الاستنتاج: قد تشير هذه الدراسة إلى أن الانتشار المرتفع لداء المشوكات الكيسية بين البشر والحيوانات في المغرب يرجع في جزء كبير منه إلى ثلاثة عوامل: 1) وفرة الكلاب 2) انخراط المجموعات السكانية المحلية في سلوكيات خطيرة و3) سوء البنية التحتية للمسالخ والممارسات المتبعة فيها. هذه الأمور لها تبعات خطيرة فيما يتعلق بالتأثير الاجتماعي الاقتصادي على المرض، وخاصة في المجتمعات الريفية الفقيرة.

Translated from English version into Arabic by Lina SM, through

TRANSLATORS WITHOUT BORDERS

摩洛哥西迪卡塞姆省囊型包虫病出现、传播、影响和防控的知识、态度和行为调查

Ikhlass el Berbri, Marie Julie Ducrotoy, Anne-Fran ϛoise Petavy, Ouaffa Fassi Fihri, Alexandra Shaw, Mohammed Bouslikhane, Franck Boue, Sue Welburn, Allal Dakkak

摘要

引言：本研究是一项针对五大被忽视的人兽共患病（NZDs）重大科研项目的一部分。本研究历时四年（2009 年 4 月至 2013 年 3 月）在西迪卡塞姆省开展包括囊型包虫病调查。

方法：以社区为单位，在 27 个公社进行问卷调查，并走访该省 10 个屠宰场，收集人兽间疾病传播的决定性因素的定性数据。即评估居民对囊型包虫病的知识、态度和行为，以及当地习俗行为对疾病传播的促进程度。严格评估屠宰场基础设施和行为及其在疾病持续传播中的作用。

结果：研究结果显示，只有半数人知晓囊型包虫病，仅 21%了解犬能传播疾病。67%的受访者表示给犬喂食不适合人类消费的反刍动物器官。在 39%的家庭中，家犬可进入家庭住宅区，包括厨房。在每户均有 1.8

只犬的社区里，犬随处可见，人犬关系则更为亲密。犬随意出入屠宰场也是一个棘手的问题，促进了疾病传播。

结论：研究表明，摩洛哥囊型包虫病的高人兽患病率主要原因有以下三个方面：1）犬只遗弃，2）当地居民的风险行为，和 3）屠宰场基础设施差，行为不规范。囊型包虫病将对社会，尤其是偏远贫困社区的经济造成严重冲击。

Translated from English version into Chinese by Chen Jin, edited by Yang Pin, through

Connaissances, attitudes et pratiques relatives à la présence, la transmission, l’impact et le contrôle de l’échinococcose cystique dans la province de Sidi Kacem, Maroc

EL BERBRI Ikhlass[1], DUCROTOY Marie Julie[3*], PETAVY Anne-Françoise[2], FASSI FIHRI Ouaffa[1], SHAW Alexandra[3,4], BOUSLIKHANE Mohammed[1], BOUE Franck[5], Sue WELBURN[3], DAKKAK Allal[1]

Résumé

Contexte : Cette étude fait partie d’un important projet de recherche mené sur cinq principales zoonoses négligées, dont l’échinococcose cystique et a été réalisée dans la province de Sidi Kacem au cours d’une période de quatre ans (avril 2009 - mars 2013).

Méthodes : Des questionnaires ont été administrés à l’échelle communautaire auprès de 27 communes au total et des visites ont eu lieu dans l’ensemble des 10 abattoirs situés dans la province afin de recueillir des données qualitatives sur les déterminants de la transmission de la maladie chez les êtres humains et les animaux. De manière plus spécifique, l’étude a permis d’évaluer les connaissances, attitudes et pratiques de la communauté liées à l’échinococcose cystique ainsi que l’étendue dans laquelle les coutumes et comportements locaux peuvent contribuer à la transmission. L’infrastructure des abattoirs et leurs pratiques ainsi que leur rôle dans la perpétuation de la transmission de la maladie ont aussi fait l’objet d’une évaluation critique.

Résultats : Les résultats démontrent que seules 50 % des personnes ont déjà entendu parler de la maladie et que parmi ces dernières, seules 21 % connaissent le rôle du chien dans la transmission de la maladie. 67 % des personnes ayant répondu au questionnaire ont affirmé que les chiens étaient nourris d’organes de ruminants supposés inaptes à la consommation humaine. Les chiens ayant un maître ont accès au foyer familial, y compris la cuisine, dans 39 % des ménages. L’étendue de cette proximité étroite entre les êtres humains et les animaux est même encore plus pertinente lorsque l’on considère l’omniprésence des chiens au sein de la communauté, avec une moyenne de 1,8 chien par ménage. L’accès non restreint des chiens aux abattoirs constitue un énorme problème qui contribue de plus à la transmission de la maladie.

Conclusion : Cette étude tend à suggérer que la prévalence élevée de l’échinococcose cystique chez l’humain et l’animal au Maroc est dans une large mesure due à trois facteurs : 1) abondance de chiens 2) adoption d’un comportement à risque par la population locale et 3) infrastructure et pratiques des abattoirs insuffisantes. Ces facteurs ont des conséquences graves en termes d’impact socio-économique de la maladie, notamment pour les communautés rurales pauvres.

Translated from English version into French by Eric Ragu, through

Осведомленность, отношение и опыт населения провинции Сиди Качем, Марокко, в вопросе распространения и влияния цистического эхинококкоса, а также контроля над ним

Ихласс ЭЛЬ БЕРБРИ[1], Мари-Жули ДЮКРОТОЙ[3*], Ан-Франсуаз ПЕТАВИ[2], Уаффа ФАССИ ФИХРИ[1], Александра Шоу[3,4], Мохаммед Буслихан[1], Франк БУ[3], Сью ВЕЛБУРН[3], Аллал ДАККАК[1]

Аннотация

История вопроса. Это исследование является частью крупного исследовательского проекта, посвященного забытым зоонотическим болезням (ЗЗБ), в том числе цистическому эхинококкосу. Исследование выполнялось в провинции Сиди Качем на протяжении четырех лет (с апреля 2009 г. по март 2013 г.)

Методы. В общине из 27 коммун было проведено анкетирование, были посещены 10 скотобоен в провинции с целью сбора качественных данных об определяющих факторах передачи болезни людям и животным. В частности, была выполнена оценка осведомленности общины, ее отношения и опыта касаемо цистического эхинококкоса; также была выполнена оценка того, насколько местные обычаи и традиции поведения могут способствовать передаче болезни. Критическому рассмотрению также подверглась структура и работа скотобоен в свете их роли в устойчивой передаче болезни.

Результаты. Согласно полученным результатам, только 50 % населения слышали о данной болезни, из них лишь 21 % знают о том, что собаки участвуют в ее передаче. По словам 67 % респондентов, собакам дают в пищу органы жвачных животных, поскольку они считаются непригодными для потребления людьми. Домашние собаки имеют доступ в дом, в том числе кухню, в 39 % домашних хозяйств. Близкое сосуществование людей и животных является еще более актуальным в свете того, что собаки присутствуют в общине повсеместно — на каждое домашнее хозяйство в среднем приходится 1,8 собаки. Серьезной проблемой является неограниченый доступ собак к скотобойням, что также способствует передаче болезни.

Вывод. Исследование показывает, что широкая распространенность цистического эхинококкуса у людей и животных в Марокко в основном объясняется тремя факторами: 1) большое количество собак; 2) рискованное поведение местного населения; 3) плохая инфраструктура и работа скотобоен. В результате действия этих факторов болезнь имеет серьезное социально-экономическое влияние, в особенности на бедное сельское население.

Translated from English version into Russian by Andriy Lapin, through

Conocimientos, actitudes y prácticas en relación con la presencia, transmisión, impacto y control de la equinococosis quística (hidatidosis) en la provincia de Sidi Kacem, Marruecos

EL BERBRI Ikhlass[1], DUCROTOY Marie Julie[3*], PETAVY Anne-Françoise[2], FASSI FIHRI Ouaffa[1], SHAW Alexandra[3,4], BOUSLIKHANE Mohammed[1], BOUE Franck[5], Sue WELBURN[3], DAKKAK Allal[1]

Resumen

Antecedentes: El presente estudio forma parte de un proyecto de investigación de mayor envergadura sobre cinco importantes zoonosis a las que no se les presta la debida atención, entre ellas, la equinococosis quística (hidatidosis), y fue realizado en la provincia de Sidi Kacem durante un período de 4 años (de abril de 2009 a marzo de 2013).

Métodos: Se distribuyeron cuestionarios a nivel de la comunidad en un total de 27 comunas, y se realizaron visitas a los 10 establecimientos de faena de la provincia, a fin de recabar datos cualitativos sobre los agentes transmisores de la enfermedad en humanos y animales. En particular, se evaluaron los conocimientos, actitudes y prácticas de la comunidad en relación con la hidatidosis, así como también cómo las costumbres y conductas locales pueden contribuir a promover su transmisión. Además, se realizó una evaluación crítica de la infraestructura y las prácticas de los establecimientos de faena y del papel que estas desempeñan en perpetuar la transmisión de la enfermedad.

Resultados: Los resultados demuestran que solo el 50% de las personas han oído hablar de la enfermedad, y que de estas, solo el 21% es consciente del papel del perro en su transmisión. 67% de quienes respondieron declararon que a los perros se los alimenta con vísceras de rumiantes consideradas inapropiados para el consumo humano. Los perros que tienen dueño tienen acceso a la casa familiar, incluida la cocina, en el 39% de los hogares. El impacto de esta estrecha proximidad entre humanos y animales es aún más relevante si se toma en cuenta que los perros son omnipresentes en la comunidad, con un promedio de 1,8 por hogar. El acceso irrestricto de los perros a los establecimientos de faena es un grave problema que contribuye a promover aún más la transmisión de la enfermedad.

Conclusión: Este estudio sugiere que la alta incidencia de la equinococosis quística (hidatidosis) en humanos y animales en Marruecos se debe fundamentalmente a tres factores: 1) la abundancia de perros; 2) la conducta de riesgo de la población local; y 3) la mala infraestructura y las prácticas inapropiadas de los establecimientos de faena. Ello tiene graves implicancias en cuanto al impacto socioeconómico, en especial para las comunidades pobres del medio rural.

Translated from English version into Spanish by Mónica Algazi, through
